# Impact of changes in waist-to-hip ratio after kidney transplantation on cardiovascular outcomes

**DOI:** 10.1038/s41598-020-80266-5

**Published:** 2021-01-12

**Authors:** Jun Gyo Gwon, Jimi Choi, Cheol Woong Jung, Chang Hun Lee, Se Won Oh, Sang-Kyung Jo, Won Yong Cho, Jae Berm Park, Kyu Ha Huh, Han Ro, Seungyeup Han, Jang-Hee Cho, Sik Lee, Jaeseok Yang, Myung-Gyu Kim

**Affiliations:** 1 Department of Transplantation and Vascular Surgery, Korea University College of Medicine, Seoul, Korea; 2 Department of Biostatistics, Korea University College of Medicine, Seoul, Korea; 3 Department of Internal Medicine, Korea University College of Medicine, Goryudae-ro 73, Seongbuk-gu, Seoul, 02841 Korea; 4 Department of Surgery, Seoul Samsung Medical Center, Sungkyunkwan University, Seoul, Korea; 5 Department of Surgery, Yonsei University College of Medicine, Seoul, Korea; 6 Department of Internal Medicine, Gachon University Gil Hospital, Incheon, Korea; 7 Department of Internal Medicine, Keimyung University School of Medicine, Daegu, Korea; 8 Department of Internal Medicine, Kyungpook National University Hospital, Daegu, Korea; 9 Department of Internal Medicine, Chonbuk National University Hospital, Jeonju, Korea; 10 Transplantation Center, Seoul National University Hospital, Seoul, Korea

**Keywords:** Cardiology, Nephrology, Urology

## Abstract

Recently, waist to hip ratio (WHR) has been reported to be a better indicator of predicting cardiovascular outcomes than body mass index (BMI). We evaluated the effects of pre or post-transplant changes of WHR or BMI on the new onset cardiovascular diseases (CVD) in recipients of kidney transplantation (KT). A total of 572 patients were enrolled from a multicenter observational cohort (KNOW-KT). Measurement of WHR and BMI was done at pre-KT, first and last visit year after KT, and the changes of these parameters and their effect on the incident CVD were analyzed. During the median follow up period of 32.73 ± 15.26 months, the new onset CVD developed in 31 out of 572 patients. The older age, diabetes mellitus and increase of WHR from pre KT or previous follow up year were found to be independent factors predicting the new onset CVD in these patients. However, baseline BMI, WHR prior to KT did not predict the incident CVD. The new metabolic burden, presented as increase of WHR in KT patients has a critical impact on the development of new onset CVD. Strategies to prevent the metabolic burden after KT might improve cardiovascular outcomes and patient’s survival.

## Introduction

Obesity is an important risk factor predicting cardiovascular disease (CVD) and mortality in the general population^1–5^. However, conflicting results have been reported in patients with non-dialysis chronic kidney disease (CKD) and those with dialysis-requiring CKD depending on the method of obesity evaluation. In particular, many reports have suggested that body mass index (BMI), a traditional indicator of obesity, is not a good predictor of CVD in patients with CKD, but that, rather, high BMI is associated with a low risk of CVD^6–9^. One of the reasons for this paradoxical relationship is that BMI, which is simply calculated using height and weight, can increase with body changes such as an increase in visceral fat or peripheral fat, and does not accurately reflect central obesity (an important risk factor of CVD). For this reason, waist-to-hip ratio (WHR) and waist circumference (WC) have been suggested as better indicators of central obesity than BMI, as predictors of coronary artery calcification (CAC) and CVD in the CKD population, and as predictors of mortality^7,10,11^. However, central obesity in patients with CKD needs to be remeasured after kidney transplantation (KT) and reevaluated for the negative effects of pretransplant obesity. This is because the effects of traditional risk factors can be altered by improvement of renal function, improvement of anemia, and new metabolic burdens after KT. Especially, improvement in water and sodium retention and nutritional status after KT may cause rapid changes in body composition and either generate new obesity or improve or worsen an underlying obesity. In addition, use of immunosuppressants such as steroids, calcineurin inhibitors, and mammalian target of rapamycin inhibitors increases the metabolic burden of hyperglycemia, hypertension, and hyperlipidemia. Therefore, it is important to systematically examine how changes in body composition affect the development and exacerbation of obesity after KT, as well as the effects of CVD. Here, we investigated the changes in BMI and WHR after KT and their impact on subsequent CVD development.

## Patients and methods

### Database and study design

This was an observational cohort study conducted at eight centers in the Republic of Korea. From July 2012 to August 2016, a total of 1080 KT recipients were enrolled in KNOW-KT (Korean Cohort Study for Outcome in Patients with Kidney Transplantation), and the present study analyzed the KNOW-KT database. KNOW-KT was registered in an international clinical trial registry (NCT02042963 at http://www.clinicaltrials.gov) on January 20th, 2014. Patients in the KNOW-KT registry were enrolled in accordance with previously described inclusion and exclusion criteria^12^. All transplants in this study were carried out under the supervision of the Korean Network for Organ Sharing (KONOS) and did not procure organ from prisoners. Of these patients, those who had a history of peritoneal dialysis before KT, had two or more KTs, a CVD history before KT or had no data on WHR were excluded from this study. Consequently, a total of 572 patients were analyzed (Supplementary Fig. 1). To confirm the impact of WHR on the incidence of CVD, the correlation between changes in WHR and the incidence of new-onset CVD after KT in these patients were analyzed. KNOW-KT was approved by the institutional review committee of each participating center (Korea University Anam Hospital, Seoul National University Hospital, Yonsei University Severance Hospital, Sungkyunkwan University Samsung Medical Center, Kyungpook National University Hospital, Chonbuk National University Hospital, Gachon University Gil Hospital, Keimyung University Dongsan Medical Center, Ulsan University Seoul Asan Medical Center) and written informed consent was obtained from all patients. Procedures performed in this manuscript are in accordance with the institutional guidelines and regulations.

**Figure 1:**
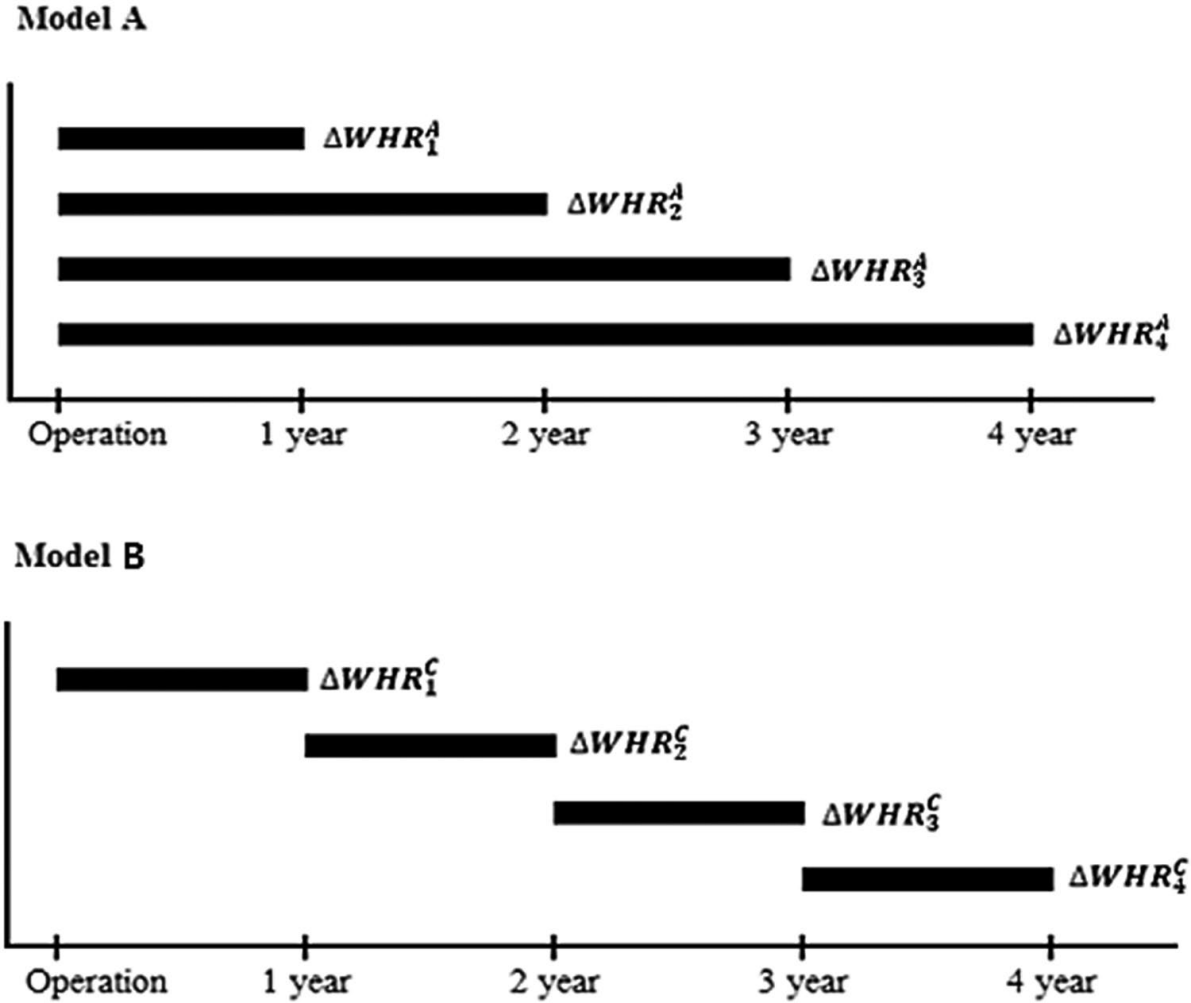
Changes in waist-to-hip ratio (WHR) every year up to n-years after kidney transplantation in patients with a cardiovascular event. The effect of WHR changes on the event occurrence was assessed. Models of patients with events up to 4 years after kidney transplantation are shown.

### Clinical variables and WHR change

CVD was evaluated as new-onset coronary artery disease, cerebrovascular disease, and peripheral arterial disease^13,14^, and the diagnosis was confirmed by the KNOW-KT registry survey conducted at each center. To improve the accuracy of the diagnosis, the specific diagnosis for each disease group was rechecked. Coronary artery disease included the diagnoses of angina pectoris, myocardial infarction, silent myocardial ischemia, and mortality due to a coronary artery problem. Cerebrovascular disease included acute neurogenic injury resulting from ischemic cerebral infarction; hemorrhagic cerebrovascular disease was excluded as it may differ from ischemic cerebrovascular disease, for which atherosclerosis is the main pathophysiologic mechanism. Peripheral arterial disease included atherosclerosis of the peripheral artery; embolism, aneurysm, or dissection of the peripheral artery were excluded. For the evaluation of central obesity, WC and hip circumference were measured, and WHR was calculated as the ratio of WC to hip circumference during baseline and subsequent periods. The changes in WHR values at annual visits were calculated using two methods: change from the pre-KT period (model A) and change from the previous-year visit (model B). A positive value of the calculated WHR change means that WHR increased over the different measurement points during the follow-up period (Fig. 1).

### Statistical analysis

Data are presented as frequency (percentage) for categorical variables and mean ± standard deviation for continuous variables. The chi-square test, Fisher’s exact test, and Student’s t-test were used, as appropriate, to compare the baseline characteristics between patients who experienced a CVD event during the follow-up period and those who did not. We calculated the incidence rate of CVD by dividing the number of events by the total person-years and multiplying by 100 for patients who had not experienced CVD before baseline. To evaluate the effect of WHR changes on CVD occurrence, two Cox proportional regression models (models A and B) were built according to two measures of WHR changes, and included other potential confounders such as age, sex, type of donor, dialysis before transplantation, smoking, comorbidities (diabetes mellitus, hyperlipidemia, hypertension), and use of immunosuppressants (calcineurin inhibitors, steroids). We applied the counting processes to all Cox proportional regression models to consider WHR changes measured at every year as a time-dependent covariate. The effects of WHR change and covariates on the CVD incidence are presented as adjusted hazard ratios and 95% confidence intervals (CIs). The hazard function for CVD according to WHR changes was smoothed using penalized spline regression and graphically presented as relative rates with 95% CIs. All statistical analyses were performed using SAS software version 9.4 (SAS Institute Inc., Cary, NC, USA) and R software version 3.6.1 (R Foundation for Statistical Computing, Vienna, Austria). A two-sided *p* value of < 0.05 was considered statistically significant.

## Results

### Patients’ characteristics

Table 1 shows the patients’ characteristics. The mean age was 44.9 ± 11.7 years, and 61.7% were men. Living- and deceased-donor transplantations were performed in 486 (84.9%) and 86 (15%) cases, respectively. Diabetes, hypertension, and hyperlipidemia were diagnosed before KT in 138 (24.1%), 524 (91.6%), and 350 (61.2%) patients, respectively. The preoperative mean WHR was 0.89 ± 0.06, and the mean BMI was 22.7 ± 3.4 kg/m^2^. Recipients received steroid (571, 99.8%) and tacrolimus (547, 95.6%) as a maintenance immunosuppressant after KT (Table 1).

**Table 1:** Baseline characteristics of the study patients before kidney transplantation.

	Total (n = 572)	New-onset CVD event (n = 31)	No CVD event (n = 541)	*p* value^a^
Women	219 (38.3)	9 (29.0)	210 (38.8)	0.276
Age (years)	44.9 ± 11.7	52.7 ± 9.2	44.5 ± 11.7	< 0.001
**Donor**				0.107
Living	486 (85.0)	24 (77.4)	462 (85.4)	
Deceased	86 (15.0)	7 (22.6)	79 (14.6)	
Hemodialysis before kidney transplantation	425 (74.3)	26 (83.9)	399 (73.8)	0.210
**Cause of chronic kidney disease**				0.037
Diabetes mellitus	132 (23.1)	14 (45.2)	118 (21.8)	
Hypertension	126 (22.0)	3 (9.7)	123 (22.7)	
Glomerulonephritis	164 (28.6)	6 (19.3)	158 (29.2)	
Polycystic kidney disease	36 (6.3)	2 (6.5)	34 (6.3)	
Others	114 (19.9)	6 (19.3)	108 (20.0)	
**Comorbid conditions**				
Smoking, ever	268 (46.9)	14 (45.2)	254 (47.0)	0.846
Diabetes mellitus	138 (24.1)	15 (48.4)	123 (22.7)	0.001
Hyperlipidemia	350 (61.2)	18 (58.1)	332 (61.4)	0.714
Hypertension	524 (91.6)	29 (93.6)	495 (91.5)	> 0.999
**Immunosuppressant**				
Calcineurin inhibitor				> 0.999
Tacrolimus	547 (95.6)	30 (96.8)	517 (95.9)	
Cyclosporin	23 (4.0)	1 (3.2)	22 (4.1)	
Steroid	571 (99.8)	31 (100.0)	540 (99.8)	> 0.999
Body mass index	22.7 ± 3.4	23.2 ± 2.6	22.6 ± 3.5	0.359
Waist-to-hip ratio	0.89 ± 0.06	0.89 ± 0.06	0.89 ± 0.07	0.994

Values are provided as n (%) or mean ± standard deviation.

CVD includes coronary artery disease, cerebrovascular disease, and peripheral arterial disease.

^a^*p* value from the chi-square test, Fisher’s exact test, or Student’s t-test, performed as appropriate.

### Post-transplant changes of WHR or BMI

After transplantation, BMI significantly decreased at 1 year from 22.8 ± 3.5 to 22.6 ± 3.2 kg/m^2^ (*p* = 0.002), but increased again thereafter and continued to increase until 5 years after KT from 22.6 ± 3.2 to 23.4 ± 3.1 kg/m^2^. However, WHR slightly decreased at 1 year after KT from 0.888 ± 0.064 to 0.884 ± 0.062 (*p* = 0.070), and the change was insignificant after 1 year (Supplementary Table 1). Changes in these parameters may reflect a correction of fluid imbalance and a rapid change in body weight due to improvement of renal function during the first year; however, thereafter, the patient’s nutritional status, medications, and metabolic burden individually affect the body composition.

### Impact of changes in WHR or BMI on new-onset CVDs

**Figure 2:**
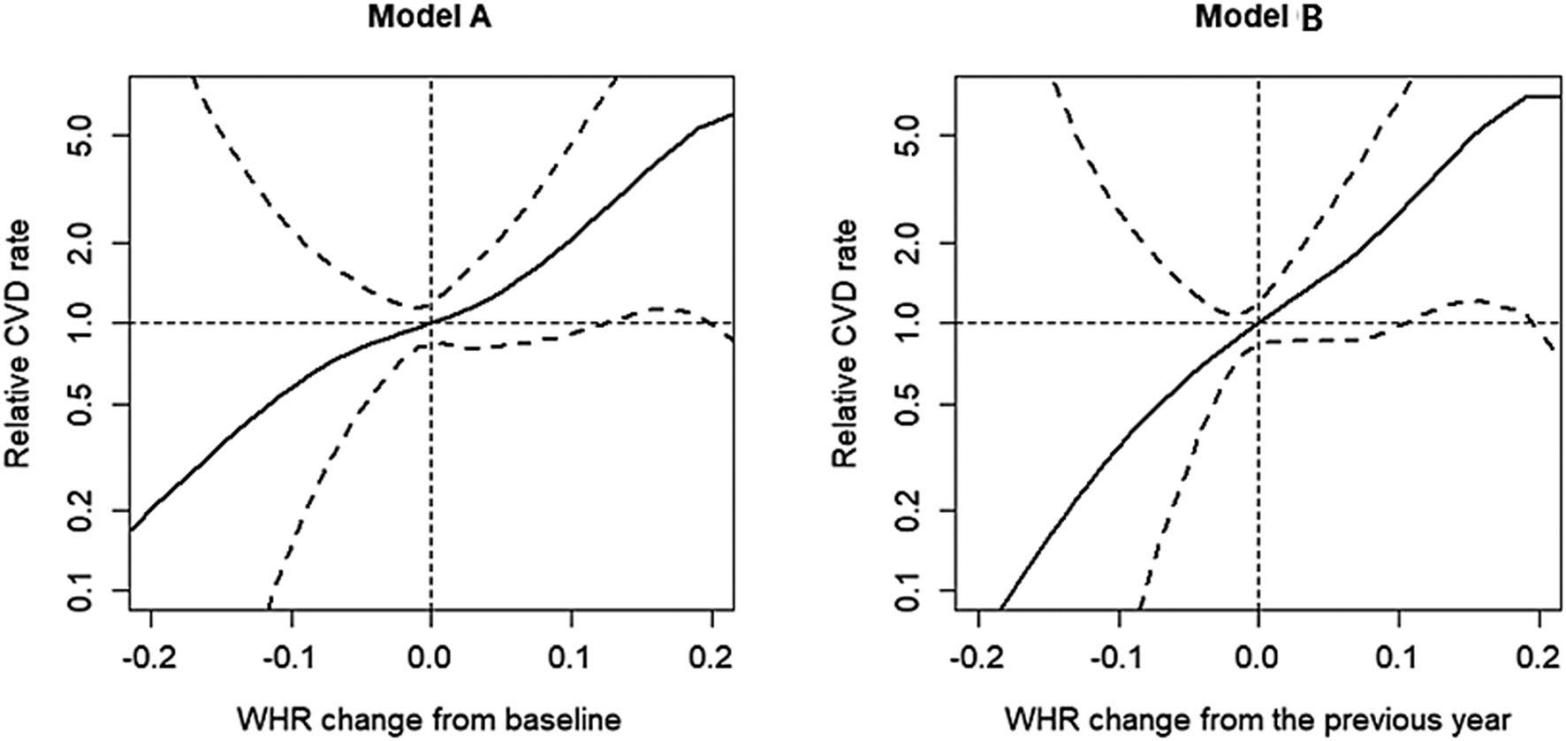
Relative hazard ratio of new-onset cardiovascular disease (CVD) according to changes in waist-to-hip ratio (WHR) (model A and model B).

**Figure 3:**
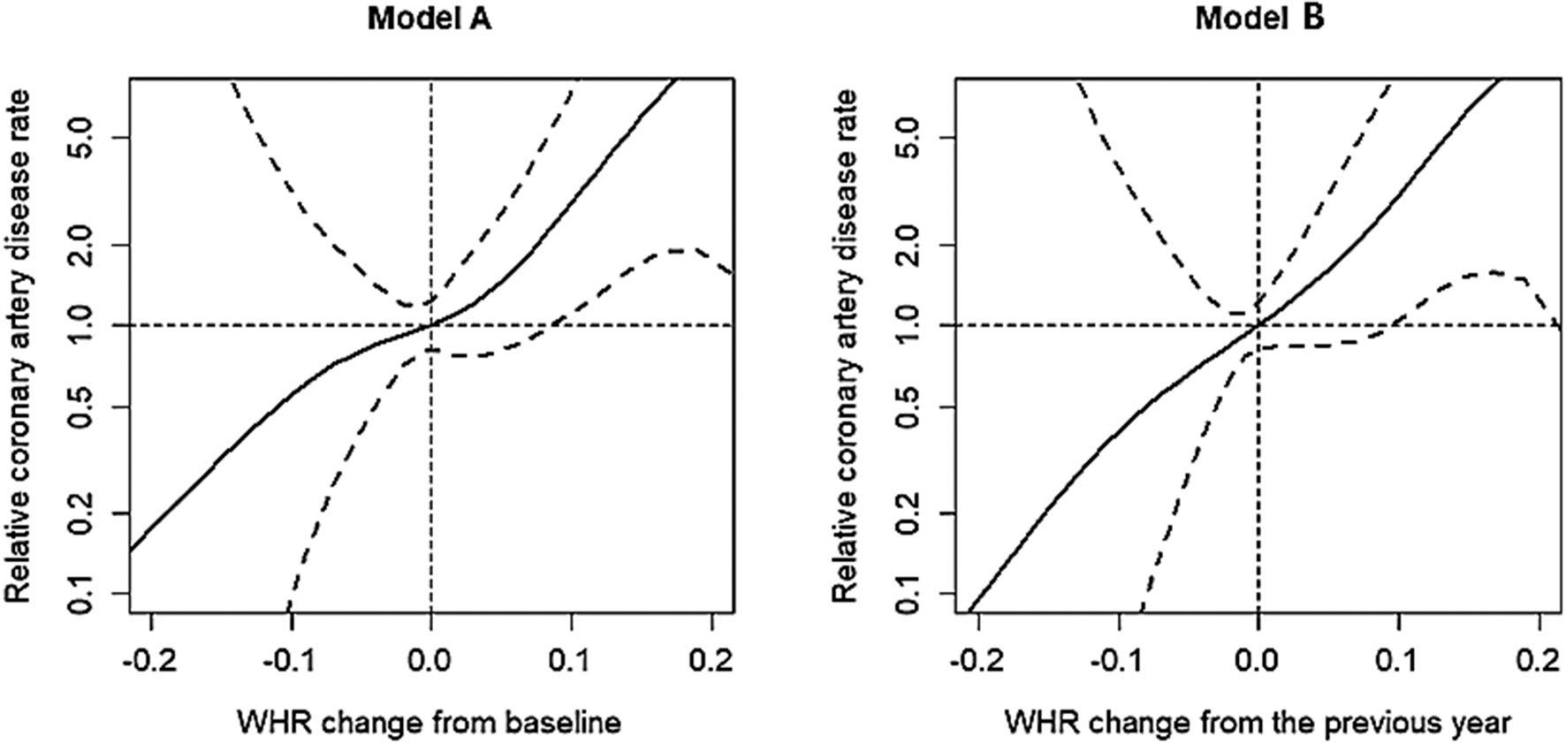
Relative hazard ratio of new-onset coronary artery disease according to changes in waist-to-hip ratio (WHR) (model A and model B).

During the follow-up, CVD newly occurred in a total of 31 patients. Coronary artery disease occurred in 20 patients, peripheral arterial disease in eight patients, and cerebrovascular disease in seven patients. As shown in Table 2, the incidence rate per 100 person-years for new-onset CVD was 2.38, and coronary artery disease was the most common CVD condition. Patients who had new-onset CVD were older (52.7 ± 9.2 vs. 44.5 ± 11.7 years, *p* < 0.001) and more often had diabetes (15 [48.4%] vs. 123 [22.7%], *p* = 0.001) than those without CVD. However, there were no significant differences in the pre-KT BMI (23.2 ± 2.6 vs. 22.6 ± 3.5 kg/m^2^, *p* = 0.359) and WHR (0.89 ± 0.06 vs. 0.89 ± 0.07, *p* = 0.994) between patients with new-onset CVD and those without. Multivariate analysis was performed to identify pre- and post-KT risk factors predicting newly developed CVD after KT. In model A, increased WHR from the pre-KT period, old age, and diabetes mellitus were risk factors of new-onset CVD (*p* = 0.012, 0.002, and 0.037, respectively). In model B, increased WHR from the previous follow-up year and old age were also significant risk factors of CVD (*p* = 0.003 and 0.003, respectively) (Table 3). The cubic spine curve was plotted using models A and B showed a trend of increasing CVD incidence as WHR increased. In particular, the incidence of CVD significantly increased when WHR in model A increased from 0.13 to 0.19 compared with pre-KT, and when WHR in model B increased from 0.11 to 0.19 compared with the previous follow-up year (Fig. 2). Subgroup analysis was performed on coronary artery disease, which had the highest incidence among the CVD conditions. Increased WHR and diabetes mellitus were significant risk factors of new-onset coronary artery disease in models A and B (*p* = 0.001 and 0.005, respectively, in model A; *p* = 0.001 and 0.0005, respectively, in model B), (Table 4). The cubic spine curve also showed a trend of increasing coronary artery disease incidence as the WHR increased, and the incidence of coronary artery disease significantly increased when the WHR change reached > 0.09 in model A and > 0.1 in model B (Fig. 3). Whether changes in BMI affected the new-onset CVD events was also analyzed. Using the same method as that for WHR change, the BMI change was analyzed using two models (A and B). In multivariate analysis, the BMI change in all two models did not affect new-onset CVD (*p* = 0.311 and 0.114, respectively) (Supplementary Table 2).

**Table 2:** Incidence of new-onset cardiovascular disease including coronary artery disease, cerebrovascular disease, and peripheral vascular disease.

Events	New-onset CVD	New-onset coronary artery disease	New-onset peripheral arterial disease	New-onset cerebrovascular disease
No. of events	31	20	8	7
Total person-years	1300.5	1358	1158.5	1119
Incidence rate per 100 person-years	2.38	1.47	0.69	0.63

New-onset CVD includes coronary artery disease or cerebrovascular disease or peripheral arterial disease.

**Table 3:** Multivariate analysis of the incidence of new-onset cardiovascular disease.

	Model A			Model B		
	Adjusted HR (95% CI)		*p* value	Adjusted HR (95% CI)		*p* value
WHR change (per 0.1)	2.28 (1.20, 4.35)		0.012	2.74 (1.43, 5.27)		0.003
Age (per 1 year)	1.06 (1.02, 1.10)		0.002	1.06 (1.02, 1.10)		0.003
Female sex	0.70 (0.28, 1.80)		0.462	0.68 (0.27, 1.74)		0.423
Living donor	0.99 (0.50, 1.97)		0.974	0.98 (0.49, 1.96)		0.952
Hemodialysis before kidney transplantation	1.42 (0.54, 3.77)		0.482	1.42 (0.53, 3.76)		0.485
Smoking, ever	0.80 (0.34, 1.89)		0.615	0.78 (0.33, 1.85)		0.577
Diabetes mellitus	2.24 (1.05, 4.79)		0.037	2.31 (1.08, 4.94)		0.031
Hyperlipidemia	0.89 (0.43, 1.84)		0.760	0.89 (0.43, 1.83)		0.747
Hypertension	0.53 (0.14, 2.06)		0.361	0.52 (0.14, 2.04)		0.351
Calcineurin inhibitor	0.92 (0.16, 5.12)		0.922	0.98 (0.17, 5.47)		0.977

WHR changes at the nth year in each model were defined as follows: model A: WHR at the nth year—WHR at baseline. model B: WHR at the nth year—WHR at the (n − 1)th year.

**Table 4:** Multivariate analysis of the incidence of new-onset coronary artery disease.

	Model A			Model B		
	Adjusted HR (95% CI)		*p* value	Adjusted HR (95% CI)		*p* value
WHR change (per 0.1)	2.95 (1.52, 5.71)		0.001	3.08 (1.57, 6.01)		0.001
Age (per 1 year)	1.06 (1.01, 1.12)		0.022	1.06 (1.01, 1.12)		0.022
Female sex	0.92 (0.27, 3.08)		0.892	0.89 (0.27, 2.98)		0.850
Living donor	1.57 (0.75, 3.30)		0.232	1.57 (0.75, 3.28)		0.235
Hemodialysis before kidney transplantation	1.27 (0.36, 4.47)		0.710	1.23 (0.35, 4.33)		0.746
Smoking, ever	0.88 (0.29, 2.74)		0.830	0.85 (0.28, 2.62)		0.777
Diabetes mellitus	4.27 (1.56,11.68)		0.005	4.25 (1.56,11.53)		0.005
Hyperlipidemia	0.65 (0.27, 1.61)		0.356	0.65 (0.26, 1.60)		0.347
Hypertension	1.65 (0.09, 31.70)		0.742	1.58 (0.08, 30.40)		0.761
Calcineurin inhibitor	0.62 (0.10, 3.87)		0.613	0.61 (0.10, 3.81)		0.600

WHR changes at the nth year in each model were defined as follows: model A: WHR at the nth year—WHR at baseline. model B: WHR at the nth year—WHR at the (n − 1)th year.

## Discussion

In the general population, obesity is an important risk factor of CVD, including left ventricular hypertrophy, heart failure, coronary artery disease, and cerebrovascular disease, and is also a predictor of mortality^1,3–5^. However, studies on obesity and CVD in patients with CKD have produced conflicting results. Many epidemiologic studies have demonstrated that in patients with predialysis CKD and those with dialysis-requiring CKD, obesity is associated with low mortality, and this relationship is referred to as the “obesity paradox”^6–9,15^. Further, a recent large cohort study showed that high BMI was associated with worse outcomes in patients with early-stage CKD; however, this association was attenuated in those with advanced-stage CKD^9^. This means that in patients with advanced-stage CKD with many comorbid conditions, the benefit of better nutritional status can be greater than the negative metabolic effect of obesity, and the presence of a low metabolic rate in obese individuals may affect the production of uremic waste, leading to better tolerance of CKD-related morbidity.

An interesting question arises about how this paradoxical effect of obesity on CKD changes when renal function is restored after KT. Several studies in transplant patients have demonstrated that obesity is associated with the development of delayed graft function after KT, and a meta-analysis reported that obese recipients have only a slightly increased risk of graft loss and experience similar survival to recipients with normal BMI^16–18^. However, the impact of pretransplant obesity on graft and recipient outcomes is still controversial because the effect of pretransplant obesity on cardiovascular health can be altered by various compounding factors due to the near-normal graft function after KT. Therefore, for transplant patients, it is necessary to reevaluate changes in obesity during the follow-up period. Recently, the impact of post-KT BMI changes on recipient outcomes was reported in large transplant cohorts^19,20^. The studies assessed BMI changes during the first year after KT when renal function, water balance, and nutritional status rapidly improved. With respect to CVD risk, a cohort consisting of Asian transplant patients showed that consistently high BMI was an important factor predicting new-onset CVD, but increase in BMI was not^20^. However, as changes in body weight and BMI during the first year of KT may not accurately reflect long-term metabolic burdens, it may be important to monitor changes in body composition for more than a year and evaluate their impact. Moreover, recent studies have shown that BMI may not be a good surrogate marker for CVD in obese patients. Because BMI is susceptible to the effects of muscle mass, fluid balance, and excess fat, it may not well reflect central obesity. As an alternative to BMI, WHR has been suggested as an index of metabolic burden in obese patients. A large-scale cohort study on patients with CKD reported that BMI was not associated with CAC, but WHR was independently associated with CAC^10^. In the study, normal BMI but higher WHR posed the highest risk of CAC, which suggests that patients who had normal BMI with less muscle mass or less subcutaneous fat but had high WHR with more visceral fat showed the highest risk for CAC. A recent study on transplant patients also found that WHR is a more sensitive predictor of clinical CVD risk than BMI^21^. However, the study was conducted in a relatively small number of pediatric transplant patients with a low CVD risk, and hyperlipidemia and even hypertension were included as CVD outcomes. Further, the effect of baseline WHR was analyzed without considering changes in body composition after KT.

Our study is the first to report the effect of pre and post-KT WHR changes on CVD outcomes. First, we analyzed changes in BMI and WHR after KT. Interestingly, only BMI significantly decreased in the first year and increased again thereafter. This reflects the rapid improvement in water and sodium balance and changes in body weight in the presence of near-normal renal function early in the transplantation period, and also means that BMI may not accurately reflect central obesity. However, WHR showed the highest increase at 1–2 years after KT and continued to slightly increase thereafter. This was considered to represent a critical time when metabolic burdens due to factors such as immunosuppressant use and improved nutritional status cause an increase in central obesity.

In general, Asians are less obese than their Western counterparts, and our study included patients with mild obesity (near-normal baseline BMI of 22.8 and WHR of 0.888). We found that even in patients with little obesity, increase of WHR can significantly affect the occurrence of CVD. In particular, analysis of models A and C showed that when the WHR change increased by about ≥ 0.1, the CVD incidence rate increased. In addition, increase in WHR was a stronger risk factor for coronary artery disease than old age and diabetes mellitus, which are important risk factors of CVD, and the risk of coronary artery disease significantly increased even with a WHR increase of 0.09. Therefore, exacerbation of WHR in a posttransplant patient with new metabolic burdens was found to be a highly important risk factor for new-onset CVD. In our study, although the change in BMI was greater than the change in WHR, BMI had a poor association with CVD. This could be because, as previous studies have shown, BMI does not reflect visceral fat deposition associated with systemic inflammation, oxidative stress, insulin resistance, and metabolic burden. Conversely, the increase in post-KT WHR rather than baseline WHR was a strong risk factor of CVD, which suggests that the new metabolic risks caused by the posttransplant nutrition and medications can be more important than pre-transplant factors for cardiovascular health in post-KT patients and increase in WHR is an important indicator.

Our study has several limitations. Grafts implanted in the abdomen after KT may affect the WC. This may be why WHR did not significantly change in the first year. However, we excluded patients with peritoneal dialysis who may have had significantly changed WC, and assessed the effect of serial changes in WHR by measuring WHR every year after KT. Nevertheless, it is possible that changes in WC before and after the transplantation offset the impact of changes in WHR from the baseline value on new-onset CVD. Second, various factors such as allograft dysfunction or steroid dose, concentration of calcineurin inhibitor, cardioprotective drugs (angiotensin converting enzyme inhibitor, antiplatelet drugs, statin, spironolactone, etc.), posttransplant infection or rejection, and economic status may affect the occurrence of CVD; however, these factors have not been analyzed in detail in the present study. Despite these limitations, the strength of this study is that, in the analysis involving traditional risk factors (DM, HTN, hyperlipidemia, smoking, etc.) for CVD, an increase in WHR that reflects central obesity was a strong independent risk for new onset CVD.

In conclusion, the paradoxical effect of obesity on patients with advanced CKD could be reestablished in the new metabolic environment after KT, and newly added cardiovascular risk factors, especially central obesity, may have significant effect on recipient survival, since CVD is a major cause of death in KT recipients with a functioning graft^22^. Therefore, monitoring and controlling the increase of WHR can be an important strategy to optimize cardiovascular outcomes and improve patient survival after KT.

## Supplementary Information

Supplementary information.

## Abbreviations

BMI: Body mass index
CAC: Coronary artery calcification
CKD: Chronic kidney disease
CVD: Cardiovascular disease
KT: Kidney transplantation
WC: Waist circumference
WHR: Waist-to-hip ratio
