# Supplementary material for: Impact of changes in waist-to-hip ratio after kidney transplantation on cardiovascular outcomes
Source: Sci Rep. 2021 Jan 12;11:783. doi: 10.1038/s41598-020-80266-5 (PMC7803733; doi:10.1038/s41598-020-80266-5)
Supplement: Supplementary file 1 — Supplementary information. [file 41598_2020_80266_MOESM1_ESM.docx]

**Impact of changes in waist-to-hip ratio after kidney transplantation on cardiovascular outcomes**

Jun Gyo Gwon^1^†, Jimi Choi^2^†, Cheol Woong Jung^1^, Chang Hun Lee^1^, Se Won Oh^3^, Sang-Kyung Jo^3^, Won Yong Cho^3^, [Jae Berm Park](https://pubmed.ncbi.nlm.nih.gov/?sort=date&term=Lee+KW&cauthor_id=32402452)^4^, Kyu Ha Huh^5^, Han Ro^6^, Seung-Yeup Han^7^, Jang-Hee Cho^8^, [Sik Lee](https://pubmed.ncbi.nlm.nih.gov/?sort=date&term=Lee+S&cauthor_id=31477423)^9^, Jaeseok Yang^10^, Myung-Gyu Kim^3^

^1^Department of Transplantation and Vascular Surgery, Korea University College of Medicine, Seoul, Korea

^2^Department of Biostatistics, Korea University College of Medicine, Seoul, Korea

^3^Department of Internal Medicine, Korea University College of Medicine, Seoul, Korea

^4^Department of Surgery, Sungkyunkwan University, Seoul Samsung Medical Center, Seoul, Korea

^5^Department of Surgery, Yonsei University College of Medicine, Seoul, Korea

^6^Department of Internal Medicine, Gachon University Gil Hospital, Incheon, Korea

^7^Department of Internal Medicine, Keimyung University College of Medicine, Daegu, Korea

^8^Department of Internal Medicine, Kyungpook National University Hospital, Daegu, Korea

^9^Department of Internal Medicine, Chonbuk National University Hospital, Jeonju, Korea

^10^Transplantation Center, Seoul National University Hospital, Seoul, Korea

**Supplementary Figure 1.** Flowchart of the study population.


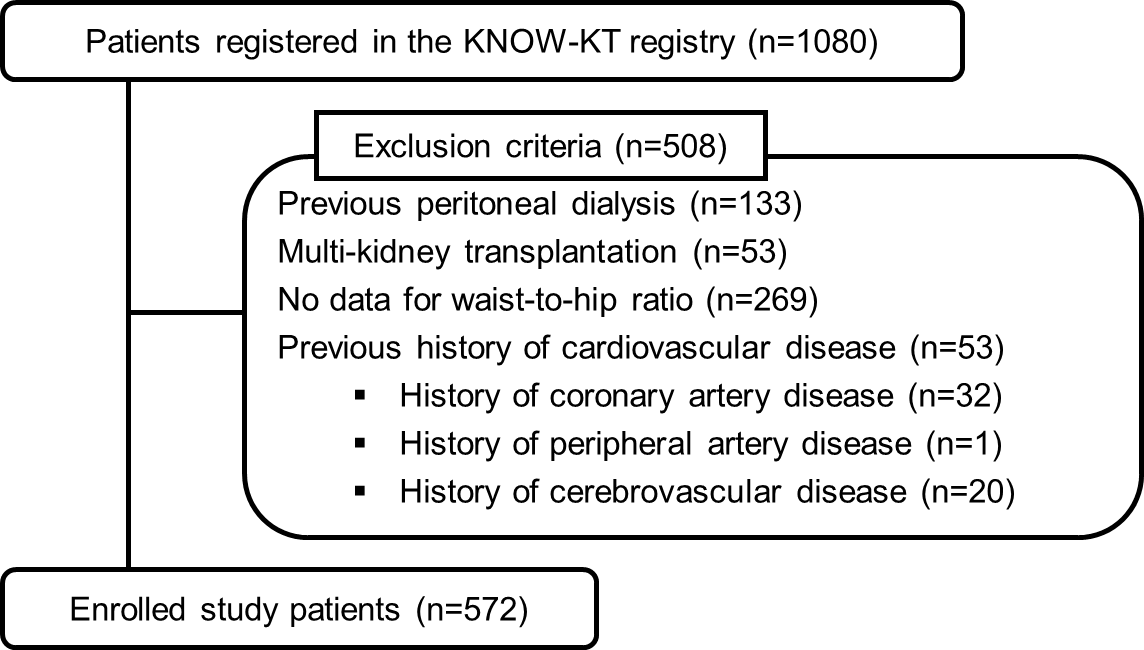


Supplementary Table 1. Changes of BMI and WHR

|  | BMI | | | |  |  | p-Value |  | WHR | | | | p-Value |
| --- | --- | --- | --- | --- | --- | --- | --- | --- | --- | --- | --- | --- | --- |
| Period | n | Mean | SD | Median | Max | Min |  |  | n | Mean | SD | Median |  |
| Baseline | 625 | 22.8 | 3.5 | 22.7 | 14.7 | 35.7 |  |  | 625 | 0.888 | 0.064 | 0.89 |  |
| Follow-up at 1 year | 583 | 22.6 | 3.2 | 22.4 | 14.1 | 33.7 |  |  | 625 | 0.884 | 0.062 | 0.89 |  |
| Follow-up at 2 years | 522 | 23.0 | 3.3 | 22.8 | 14.2 | 37.2 |  |  | 506 | 0.886 | 0.060 | 0.89 |  |
| Follow-up at 3 years | 396 | 23.2 | 3.2 | 23.1 | 15.6 | 35.4 |  |  | 353 | 0.885 | 0.060 | 0.89 |  |
| Follow-up at 4 years | 231 | 23.3 | 3.1 | 23.3 | 15.8 | 34.5 |  |  | 215 | 0.884 | 0.060 | 0.88 |  |
| Follow-up at 5 years | 67 | 23.4 | 3.1 | 23.8 | 16.5 | 30.0 |  |  | 65 | 0.895 | 0.067 | 0.89 |  |
| Compared with baseline |  |  |  |  |  |  |  |  |  |  |  |  |  |
| Follow-up at 1 year | 583 | -0.25 | 1.94 | -0.30 | -7.3 | 6.5 | 0.002 |  | 625 | -0.003 | 0.046 | 0.000 | 0.070 |
| Follow-up at 2 years | 522 | +0.14 | 2.09 | 0.20 | -6.9 | 8.2 | 0.132 |  | 506 | +0.003 | 0.045 | 0.000 | 0.183 |
| Follow-up at 3 years | 396 | +0.50 | 2.09 | 0.60 | -7.2 | 10.0 | <0.001 |  | 353 | +0.003 | 0.046 | 0.000 | 0.205 |
| Follow-up at 4 years | 231 | +0.50 | 1.96 | 0.50 | -4.3 | 6.3 | <0.001 |  | 215 | +0.001 | 0.050 | 0.000 | 0.733 |
| Follow-up at 5 years | 67 | +0.81 | 2.16 | 0.90 | -4.9 | 6.3 | 0.003 |  | 65 | -0.001 | 0.066 | 0.000 | 0.880 |
| Compared with  the previous year | |  |  |  |  |  |  |  |  |  |  |  |  |
| Follow-up at 1 year | 583 | -0.25 | 1.94 | -0.30 | -7.3 | 6.5 | 0.002 |  | 625 | -0.003 | 0.046 | 0.000 | 0.070 |
| Follow-up at 2 years | 509 | +0.36 | 1.27 | 0.30 | -6.1 | 6.4 | <0.001 |  | 506 | +0.004 | 0.034 | 0.000 | 0.005 |
| Follow-up at 3 years | 385 | +0.25 | 1.18 | 0.20 | -5.0 | 6.6 | <0.001 |  | 346 | +0.000 | 0.039 | 0.000 | 0.923 |
| Follow-up at 4 years | 224 | +0.06 | 1.05 | 0.00 | -4.4 | 4.9 | 0.372 |  | 214 | -0.001 | 0.025 | 0.000 | 0.401 |
| Follow-up at 5 years | 67 | +0.27 | 1.02 | 0.00 | -2.1 | 4.5 | 0.031 |  | 65 | +0.000 | 0.042 | 0.000 | 0.953 |

Supplementary Table 2. Multivariate analysis of the incidence of new-onset cardiovascular disease

|  | Model A | | | |  |  | Model B | | | | |
| --- | --- | --- | --- | --- | --- | --- | --- | --- | --- | --- | --- |
|  | Adjusted HR (95% CI) | | | p-Value |  |  | Adjusted HR (95% CI) | | | p-Value | |
| BMI change (per 1) | 1.10 | (0.91 | , 1.33) | 0.311 |  |  | 1.19 | (0.96 | , 1.48) | 0.114 |  |
| Age (per 1 year) | 1.06 | (1.02 | , 1.10) | 0.002 |  |  | 1.06 | (1.02 | , 1.10) | 0.004 |  |
| Female sex | 0.68 | (0.26 | , 1.74) | 0.416 |  |  | 0.74 | (0.28 | , 1.94) | 0.540 |  |
| Living donor | 0.96 | (0.48 | , 1.93) | 0.912 |  |  | 1.01 | (0.51 | , 2.02) | 0.976 |  |
| Hemodialysis before kidney transplantation | 1.39 | (0.52 | , 3.68) | 0.511 |  |  | 1.34 | (0.50 | , 3.58) | 0.560 |  |
| Smoking, ever | 0.85 | (0.36 | , 2.04) | 0.718 |  |  | 0.93 | (0.38 | , 2.27) | 0.879 |  |
| Diabetes mellitus | 2.21 | (1.03 | , 4.75) | 0.042 |  |  | 2.38 | (1.09 | , 5.17) | 0.029 |  |
| Hyperlipidemia | 0.87 | (0.42 | , 1.79) | 0.701 |  |  | 0.93 | (0.44 | , 1.95) | 0.842 |  |
| Hypertension | 0.47 | (0.12 | , 1.93) | 0.297 |  |  | 0.45 | (0.11 | , 1.84) | 0.267 |  |
| Calcineurin inhibitor | 0.95 | (0.17 | , 5.37) | 0.957 |  |  | 0.92 | (0.16 | , 5.17) | 0.921 |  |

BMI changes at the n^th^ year in each model were defined as follows:

Model A: BMI at the n^th^ year–BMI at baseline

Model B: BMI at the n^th^ year–BMI at the (n–1)^th^
